# Hearing and vision screening for preschool children using mobile technology, South Africa

**DOI:** 10.2471/BLT.18.227876

**Published:** 2019-06-19

**Authors:** Susan Eksteen, Stefan Launer, Hannah Kuper, Robert H Eikelboom, Andrew Bastawrous, De Wet Swanepoel

**Affiliations:** a Department of Speech-Language Pathology and Audiology, University of Pretoria, c/o Lynnwood and University Roads, Hatfield, Pretoria 0002, South Africa.; b Sonova AG, Science and Technology, Stäfa, Switzerland.; c International Centre for Eye Health, London School of Hygiene & Tropical Medicine, London, England.; d Ear Science Institute Australia, Subiaco, Australia.; Correspondence to De Wet Swanepoel (email: dewet.swanepoel@up.ac.za).

## Abstract

**Objective:**

To implement and evaluate a community-based hearing and vision screening programme for preschool children in the Western Cape, South Africa, supported by mobile health technology (mHealth) and delivered by community health workers (CHWs).

**Methods:**

We trained four CHWs to provide dual sensory screening in preschool centres of Khayelitsha and Mitchells Plain during September 2017–December 2018. CHWs screened children aged 4–7 years using mHealth software applications on smartphones. We used logistic regression analysis to evaluate the association between screening results and age, sex and test duration, and, for hearing, excessive background noise levels.

**Results:**

CHWs screened 94.4% (8023/10 362) of eligible children at 271 centres at a cost of 5.63 United States dollars per child. The number of children who failed an initial hearing and visual test was 435 (5.4%) and 170 (2.1%), respectively. Hearing test failure was associated with longer test times (odds ratio, OR: 1.022; 95% confidence interval, CI: 1.021–1.024) and excessive background noise levels at 1 kilohertz (kHz) (e.g. OR for left ear: 1.688; 95% CI: 1.198–2.377). Visual screening failure was associated with longer test duration (OR: 1.003; 95% CI: 1.002–1.005) and younger age (OR: 0.629; 95% CI: 0.520–0.761). Of the total screened, 111 (1.4%) children were diagnosed with a hearing and/or visual impairment.

**Conclusion:**

mHealth-supported CHW-delivered hearing and vision screening in preschool centres provided a low-cost, acceptable and accessible service, contributing to lower referral numbers to resource-constrained public health institutions.

## Résumé

**Objectif:**

Mettre en place et évaluer un programme de dépistage communautaire des troubles auditifs et visuels chez les enfants d'âge préscolaire dans la province du Cap-Occidental, en Afrique du Sud, effectué à l'aide de technologies médicales mobiles par des agents de santé communautaires.

**Méthodes:**

Nous avons formé quatre agents de santé communautaires à réaliser des doubles dépistages sensoriels dans des centres préscolaires de Khayelitsha et de Mitchells Plain de septembre 2017 à décembre 2018. Ces agents de santé ont examiné des enfants âgés de 4 à 7 ans à l'aide d'applications spécifiques sur smartphones. Nous avons réalisé une analyse de régression logistique pour évaluer l'association entre les résultats du dépistage et l'âge, le sexe et la durée du test ainsi que, pour le dépistage auditif, les niveaux de bruit de fond excessifs.

**Résultats:**

Les agents de santé communautaires ont examiné 94,4% (8023/10 362) des enfants éligibles dans 271 centres, pour un coût de 5,63 dollars des États-Unis par enfant. Le nombre d'enfants qui n'ont pu bénéficier d'un test initial de dépistage auditif et visuel était respectivement de 435 (5,4%) et 170 (2,1%). L'échec du test auditif était associé à une durée de test plus longue (rapport des cotes, RC: 1,022; intervalle de confiance de 95%, IC: 1,021–1,024) et à des niveaux de bruit de fond excessifs à 1 kHz (par ex., RC pour l'oreille gauche: 1,688; IC 95%: 1,198-2,377). L'échec du dépistage visuel était associé à une durée de test plus longue (RC: 1,003; IC 95%: 1,002–1,005) et à un plus jeune âge (RC: 0,629; IC 95%: 0,520-0,761). Sur le nombre total d'enfants examinés, 111 (1,4%) présentaient une déficience auditive et/ou visuelle.

**Conclusion:**

Le dépistage auditif et visuel sur mobile par des agents de santé communautaires dans des centres préscolaires a permis de proposer un service accessible, acceptable et à faible coût, qui a contribué à réduire le nombre de consultations dans les établissements de santé publics disposant de peu de ressources.****

## Resumen

**Objetivo:**

Implementar y evaluar un programa comunitario para examinar la audición y la visión de los niños en edad preescolar en el Cabo Occidental, Sudáfrica, apoyado por la tecnología de salud móvil (mHealth) e impartido por los trabajadores de la salud de la comunidad (CHW, por sus siglas en inglés).

**Métodos:**

Capacitamos a cuatro CHW para que realizaran exámenes sensoriales duales en centros preescolares de Khayelitsha y Mitchells Plain entre septiembre de 2017 y diciembre de 2018. Los CHW examinaron a niños de 4 a 7 años de edad utilizando las aplicaciones de software mHealth en teléfonos inteligentes. Se utilizó el análisis de regresión logística para evaluar la asociación entre los resultados del examen y la edad, el sexo y la duración del mismo, y, en el caso de la audición, los niveles excesivos de ruido de fondo.

**Resultados:**

Los CHW examinaron al 94,4 % (8 023/10 362) de los niños que reunían los requisitos en 271 centros, a un coste de 5,63 dólares estadounidenses por niño. El número de niños que no pasaron una prueba inicial de audición y visión fue de 435 (5,4 %) y 170 (2,1 %), respectivamente. El fallo de la prueba de audición se asoció con tiempos de prueba más largos (razón de momios, RM: 1,022; intervalo de confianza del 95 %, IC: 1,021-1,024) y niveles excesivos de ruido de fondo a 1 kHz (p.ej. RM para el oído izquierdo: 1,688; IC del 95 %: 1,198-2,377). El fallo del examen visual se asoció con una mayor duración de la prueba (RM: 1,003; IC del 95 %: 1,002-1,005) y una edad más temprana (RM: 0,629; IC del 95 %: 0,520-0,761). Del total de niños examinados, 111 (1,4 %) fueron diagnosticados con un impedimento auditivo y/o visual.

**Conclusión:**

mHealth, con apoyo de los CHW, realizó exámenes de audición y visión en los centros preescolares y proporcionó un servicio de bajo coste, aceptable y accesible, lo que contribuyó a reducir el número de remisiones a las instituciones de salud pública con recursos limitados.

## ملخص

**الغرض:**

تنفيذ وتقييم البرنامج المجتمعي لفحص السمع والبصر للأطفال في سن ما قبل المدرسة في ويسترن كيب، بجنوب أفريقيا، بدعم من التكنولوجيا الصحية عبر الهاتف المحمول (mHealth) والتي يقدمها عمال الصحة المجتمعية (CHW).

**الطريقة:**

قمنا بتدريب أربعة من عمال الصحة المجتمعية على تقديم فحص ثنائي الحواس في مراكز ما قبل سن المدرسة في خايليتشا وميتشلز بلين خلال الفترة من سبتمبر/أيلول 2017 إلى ديسمبر/كانون أول 2018. قام عمال الصحة المجتمعية بفحص الأطفال الذين تتراوح أعمارهم بين 4 و7 سنوات باستخدام تطبيقات برامج mHealth على الهواتف الذكية. استخدمنا تحليل التحوف اللوجيستي لتقييم الارتباط بين نتائج الاختبار والعمر والجنس ومدة الاختبار، وكذلك بالنسبة للسمع، تقييم مستويات الضوضاء العالية في الخلفية.

**النتائج:**

فحص عمال الصحة المجتمعية 94.4٪ (8023/10362) من الأطفال المؤهلين في 271 مركزًا بتكلفة 5.63 دولارًا أمريكيًا للطفل. كان عدد الأطفال الذين فشلوا في اختبار السمع الأولي 435 طفلاً (5.4٪)، وفي اختبار الرؤية الأولى 170 طفلاً (2.1٪)، على التوالي. ارتبط الفشل في اختبار السمع بأوقات أطول للاختبار (نسبة الاحتمالات: 1.022؛ فاصل الثقة 95٪: 1.021 إلى 1.024) وبمستويات ضوضاء عالية في الخلفية عند 1 كيلو هرتز (أي أن نسبة الاحتمالات للأذن اليسرى: 1.688؛ ‏بفاصل ثقة 95٪: 1.198 إلى 2.377).ارتبط الفشل في فحص الرؤية بمدة أطول للاختبار (نسبة الاحتمالات: 1.003؛ ‏بفاصل ثقة 95٪: 1.0021 إلى 1.005) والعمر الأصغر (نسبة الاحتمالات: 0.629؛ ‏بفاصل ثقة 95٪: 0.520 إلى 0.761).من بين إجمالي الأطفال الذين تم فحصهم، تم تشخيص 111 طفلاً (1.4٪) بأنهم يعانون من ضعف السمع و/أو ضعف الرؤية.

**الاستنتاج:**

قام عمال ****الصحة المجتمعية المدعومون بالتكنولوجيا الصحية على الهاتف المحمول، بفحص السمع والرؤية في مراكز الأطفال ما قبل سن المدرسة، وهو ما وفر خدمة منخفضة التكلفة ومقبولة ويسهل الحصول عليها، مما أسهم في خفض الأعداد المحالة إلى مؤسسات الصحة العامة محدودة الموارد. ****

## 摘要

**目的:**

由移动医疗技术 (mHealth) 支持、社区卫生工作者推广，旨在为南非西开普省的学龄前儿童实施和评估以社区为基础的听力和视力筛查计划。

**方法:**

我们培训了四位社区卫生工作者，在 2017 年 9 月至 2018 年 12 月期间为 Khayelitsha 和 Mitchells 平原的学前中心提供两项感觉器官（听力、视力）的筛查。社区卫生工作者使用智能手机上的 mHealth 应用程序对 4-7 岁的儿童进行筛查。我们使用逻辑回归分析来评估筛查结果与年龄、性别和测试持续时间之间的关联，至于听力筛查，我们还评估了其与过高的背景噪音之间的关联。

**结果:**

社区卫生工作者在 271 个中心筛查了 94.4％（8023/10362）符合条件的儿童，每名儿童的费用为 5.63 美元。初次听力和视力测试失败的儿童人数分别为 435 人（占比 5.4％）和 170 人（占比 2.1％）。听力测试失败与较长的测试时间（优势比，OR：1.022；95％ 置信区间，CI：1.021-1.024）和 1 千赫兹过高的背景噪音水平（例如左耳的 OR：1.688；95% CI：1.198-2.377）有关。视觉筛查失败与较长的测试时间（OR：1.003；95% CI：1.002–1.005）与低龄（OR: 0.629；95% CI: 0.520-0.761）有关。在筛查的总人数中，111 名（占比 1.4％）儿童被诊断出听力和/或视力障碍。

**结论:**

mHealth 支持、社区卫生工作者推广的学前中心听力和视力筛查是低成本、可接受和可获得的服务，有助于降低向资源有限的公共卫生机构的转诊人数。****

## Резюме

**Цель:**

Внедрение и оценка программы проверки зрения и слуха у детей дошкольного возраста по месту проживания в регионе Западного мыса, Южная Африка, проводимой при поддержке технологии мобильного здравоохранения (mHealth) местными медицинскими работниками (ММР).

**Методы:**

Авторы обучили четырех ММР методике проверки сенсорного восприятия по двум каналам, которая проводилась в центрах дошкольного воспитания в Хайелитше и Митчеллс Плейн в период с сентября 2017 года по декабрь 2018 года. ММР оценивали проверяемые показатели здоровья детей в возрасте от 4 до 7 лет при помощи программного приложения mHealth на смартфонах. Авторы применяли анализ с использованием методов логистической регрессии для оценки взаимосвязи между результатами проверки и возрастом, полом и длительностью теста; при проверке слуха дополнительно учитывался уровень фонового шума.

**Результаты:**

Всего силами ММР было проверено 94,4% детей (8032 из 10 362) соответствующего возраста в 271 центре; расходы составили 5,63 доллара США на одного ребенка. Количество детей, не прошедших первоначальный тест для проверки слуха и зрения, составило 435 (5,4%) и 170 (2,1%) соответственно. Невозможность пройти тест для проверки слуха ассоциировалась с большей продолжительностью теста (показатель шансов, ПШ: 1,022; 95%-й ДИ: 1,021–1,024) и сильными фоновыми шумами на уровне 1 кГц (например, ПШ для левого уха составил 1,688; 95%-й ДИ: 1,198–2,377). Невозможность пройти тест для проверки зрения была связана с большей продолжительностью теста (ПШ: 1,003; 95%-й ДИ: 1,002–1,005) и младшим возрастом (ПШ: 0,629; 95%-й ДИ: 0,520–0,761). Из общего числа детей, прошедших проверку, 111 (1,4%) был поставлен диагноз нарушения слуха и/или зрения.

**Выводы:**

Проверка слуха и зрения силами ММР при поддержке мобильного приложения mHealth в центрах дошкольного воспитания представляет собой недорогую, приемлемую и доступную услугу, которая позволяет снизить количество направлений к специалистам в учреждениях общественного здравоохранения, страдающих от нехватки ресурсов.

## Introduction

Sensory inputs of hearing and vision during early childhood development support the achievement of optimal language, speech and educational outcomes.^1^^,^^2^ Early detection of sensory impairments is essential for facilitating early childhood development, socioemotional well-being and academic success,^1^^–^^4^ as well as the sustainable development goals related to education.^5^

Hearing and vision impairments are the most common global developmental disabilities in children younger than 5 years, affecting 15.5 and 25.2 million, respectively,^6^ 95% of whom live in low- and middle-income countries.^6^^–^^8^ Services are usually unavailable or inaccessible in these countries because of an absence of systematic screening programmes for children, prohibitive equipment cost and a shortage of trained personnel.^2^^,^^9^^–^^11^ An awareness and knowledge of sensory impairments, their potential impact on a child’s development and potential rehabilitative solutions are also poor among early childhood practitioners in underprivileged communities.^12^

The evidence base on the value of community-based programmes incorporating mobile health technology (mHealth) for hearing and vision loss is growing.^13^^–^^15^ Community health workers (CHWs)^16^ play an important role in improving access to hearing services, including in screening and raising community awareness.^15^^,^^17^ mHealth has been recognized as increasingly important in supporting the achievement of the sustainable development goals^18^ and addressing access and affordability in underserved populations;^8^^,^^19^ it also has the potential to improve health system efficiency, quality of preventative care and health outcomes.^20^^,^^21^ Validated smartphone applications (apps), including automated tests for hearing and vision screening, pre-specified screening protocols for result interpretation, cloud-based data management for surveillance of programme performance and geolocation-based referral, allow CHWs to undertake decentralized screening and identify cases for referral.^8^^,^^13^^–^^15^^,^^22^^–^^24^ CHWs have reported such apps as user-friendly and efficient.^8^^,^^12^^,^^22^

The feasibility of community-based services facilitated by CHWs and supported by mHealth for hearing screening in homes and in early childhood development centres (informal day care centres for preschool children) in Gauteng, South Africa, has already been assessed.^14^^,^^15^ A model based on preschool centres is particularly relevant for low- and middle-income countries, where systematic newborn hearing screening is unavailable^25^ and school-entry screening is potentially the first point of access to services.

Continuing from these feasibility studies, we implemented an mHealth-supported screening programme in which children’s hearing and vision services were provided by CHWs in preschool centres. We describe this community-based service-delivery model and evaluate its success in terms of acceptability (consent return numbers), coverage (number of eligible children screened), quality indicators (duration of tests and number of hearing tests conducted under conditions of excessive noise levels), community-based second screening attendances and diagnostic centre referral attendances. We also discuss the challenges met during this implementation and the strategies developed to overcome these.

## Methods

### Study setting and preparation

We implemented our screening programme within the preschool centres of the partially informal townships of Khayelitsha and Mitchells Plain of the Western Cape province, South Africa, during September 2017 to December 2018.^26^ The joint population of Khayelitsha and Mitchells Plain was estimated as 702 234 in 2011, including 61 094 children aged 5–9 years.^27^ Most are not native English speakers.^27^ The majority (97.0%; 181145/186803) of households within the study area are classified as low- and middle-income, with 15.7% (29408/186803) having no income.^27^

Before implementation, we conducted a situational analysis of the potential referral routes to hearing and vision services and established follow-up pathways. We tested and finalized a simplified one-page consent form and screening protocols. We formed partnerships with local non-profit organizations supporting the preschool centres in the community and introduced the screening programme via the quarterly symposiums of preschool centre principals.

### Appointment of CHWs

We appointed four CHWs to conduct the combined sensory screening across all preschool centres within the study area. We placed an advertisement on notice boards within the community and conducted interviews with candidates. The four CHWs (one project administrator/screener and three screeners) were appointed on a contract basis for the duration of the programme and were paid a monthly salary. Members of the community themselves, these CHWs had a deep understanding of relevant cultural beliefs and biases regarding health services and sensory impairments. None of the CHWs had received any formal training on hearing or vision health care previously.

The audiologist managing the project delivered a 5-day training course to the CHWs on hearing and vision theory, the screening process, observation of screening in the field, practical training on using the equipment and assessment of a child’s responses. The course was held at the Carel du Toit Centre, Cape Town, South Africa, the site of the project implementation partner and employer of the audiologist. The course delivery costs were included in the project management fee. CHWs performed initial screening under supervision. The project manager chaired weekly meetings at the Carel du Toit Centre with the CHWs, allowing for further training based on any queries.

### Implementation

We mapped all preschool centres (facility name, geolocation and contact person) within the study area using the facility-mapping feature of the mobile platform and invited principals to sign a participation agreement. Within the participating centres, the parents of attending children (4–7 years) indicated their agreement to be included in the study by returning a signed consent form. To increase accessibility, we provided the parent or caregiver with the option to complete the form either in English or in their native language. CHWs distributed posters and leaflets within the preschool centres, emphasized the importance of hearing for learning to centre staff and shared information on the risk factors and signs of hearing loss.

Using mHealth, CHWs performed hearing and vision screening of all children who returned signed consent forms at their respective preschool centres during the 265 screening days held over the 16-month period. The amount of time spent on screening at a particular preschool centre depended upon its size. At any one centre, screening was usually available for some portion of a single day up to a maximum of 2 days at a date agreed in advance with the preschool principal. CHWs performed an immediate rescreen if a child failed the first screening test. Screening results were automatically sent to the child’s parent or caregiver via text message through the mHealth cloud platform. In the case of no available contact number, parents had access to the project administrator’s number and could send a free text to the project administrator, requesting a telephone call with the results.

Children who failed the initial hearing screening (at 25 decibel [dB] hearing level at 1, 2 and 4 kilohertz [kHz]) and rescreening (at 25 dB hearing level at the frequencies at which the child failed the initial test) received a community-based second screening (at 0.5–8 kHz) 1 week later at their preschool, including otoscopy. The project audiologist conducted this second screening, enabling the CHWs to continue with their schedule of initial screenings. Children who failed this second screening were referred to public health diagnostic audiology services. Children who failed the initial vision screening and rescreening (a visual acuity of less than 0.3 LogMAR (logarithm of minimum angle of resolution) in both eyes, or less than 0.4 LogMAR in one eye regardless of acuity in the other eye) were referred to primary health care facilities for a diagnostic optometric evaluation.

Parents were informed about their child’s referral by letter and reminded by telephone the day before the diagnostic evaluation. All follow-up services and interventions were provided by public health services, for example, hearing aids, spectacles or other medical intervention. CHWs kept a record of all costs incurred and challenges encountered and provided feedback to the project manager who tracked results and outcomes.

### Technology

The mHealth technology platform (hearX Group, Pretoria, South Africa) synchronizes patient results between the cloud and the smartphone software. The smartphones host point-of-care hearing and vision screening apps. We used the mHealth evidence reporting and assessment checklist to review and report on our mHealth-supported programme.^21^

CHWs used the hearScreen app (hearX Group) on a Samsung A3 smartphone with the operating system Android version 8.0 (Google, Mountain View, United States of America), connected to supra-aural Sennheiser HD280 headphones (Sennheiser, Wedemark, Germany) that had been calibrated according to prescribed standards (International Organization for Standardization, ISO 389–1).^28^ We calibrated the app to monitor environmental noise with the smartphone microphone.^14^^,^^23^^,^^24^ Children who failed the initial screen and immediate rescreen were referred to a second screening, at which children were tested via the validated hearTest app^29^ for threshold testing on the same device across a wider range of frequencies (0.5–8 kHz).

The publicly available Peek Acuity application (Peek Vision, London, United Kingdom) was used to screen visual acuity on the same smartphone. This test follows the standard Early Treatment Diabetic Retinopathy Study chart design, using a Tumbling E optotype, and is capable of acuity measurements consistent with test–retest variability of acuities measured using 5-letters-per-line retro-illuminated LogMAR charts.^8^

Data collected by the smartphone were uploaded to the cloud storage through mobile telephone networks at the end of each test.^23^^,^^24^ We ensured the security of the mHealth app and server through use of local data encryption at rest using Advanced Encryption Standard 256 bit. We secured authentication with the server via the use of Secure Sockets Layer connections. We ensured that access to smartphone and cloud-based data were protected by user password.

### Data collection and analysis

We extracted data from the secure cloud-based server to an Excel (Microsoft, Redmond, USA) spreadsheet for statistical analysis using Statistical Package for the Social Sciences software (IBM, Armonk, USA). Using Excel, we recorded and quantified test outcomes (pass or fail), test durations and the numbers being referred to and attending second screenings and diagnostic centres. We used logistic regression analysis to evaluate the association between screening outcome and age, sex and test duration for both vision and hearing screening; for hearing, we also evaluated the association between test outcome and excessive noise levels at each frequency. Significance was set at *P* < 0.05.

### Ethical considerations

Ethical clearance was obtained from the Research Ethics Committee of the Faculty of Humanities of the University of Pretoria on 4 October 2017 (GW20170922HS).

## Results

**Fig. 1:**
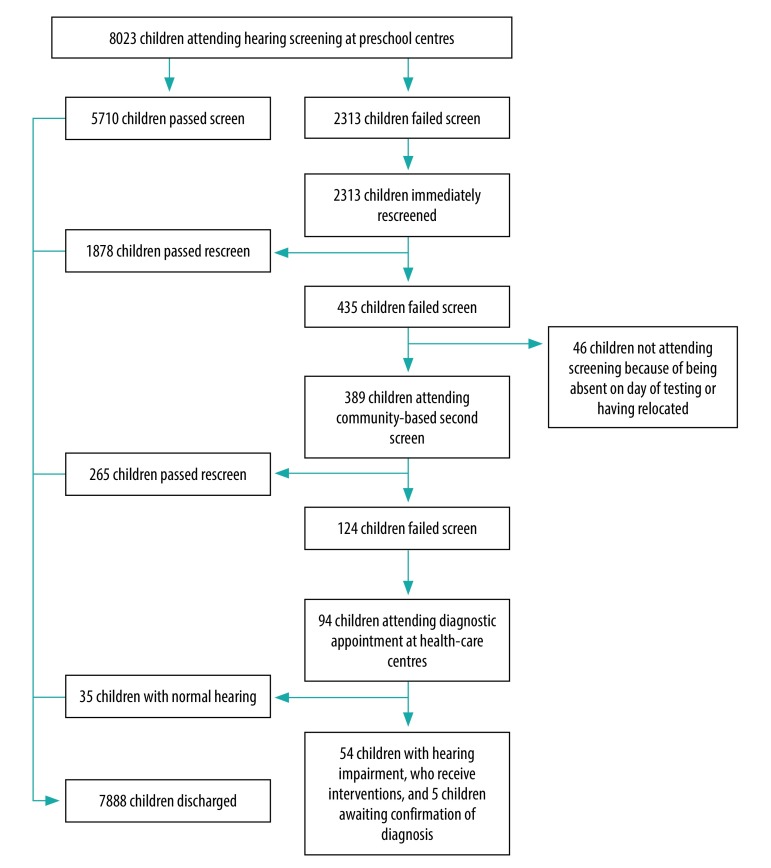
mHealth-supported community-based screening for hearing impairment, South Africa, September 2017–December 2018

**Fig. 2:**
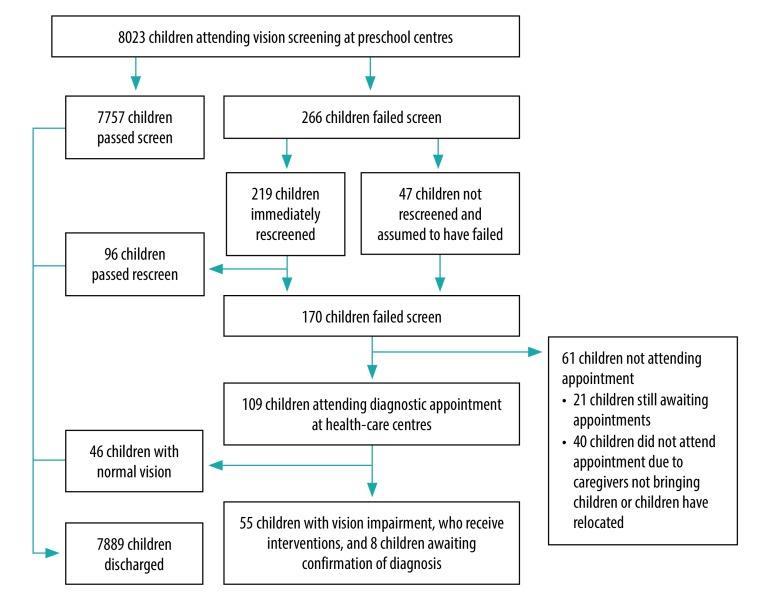
mHealth-supported community-based screening for visual impairment, South Africa, September 2017–December 2018

The 271 preschool centres participating in our study included a total of 10 362 children. Signed consent forms were returned for 8497 (82.0%) of these children and 8023 (94.4%) of eligible participants were in attendance on screening days to undergo hearing and visual screening (Table 1; Fig. 1 and Fig. 2). One in three (32.3%) parents completed the consent form in their mother tongue as opposed to English. An average of 500 children were screened each month, at a cost of 5.63 United States dollars per child (Table 2).

**Table 1:** Children screened for hearing and visual impairment via mHealth-supported community-based programme, South Africa, September 2017–December 2018

Outcome	Children screened *n* = 8023		
	Hearing impairment	Visual impairment	Both hearing and visual impairment
**No. (%) who failed initial screening**	2313 (28.8)	266 (3.3)	58 (0.7)
**No. (%) who failed immediate rescreen**	435 (5.4)	170 (2.1)^a^	19 (0.2)
Of 3972 boys	205 (5.2)	84 (2.1)	10 (0.3)
Of 4051 girls	230 (5.7)	86 (2.1)	9 (0.2)
Of 1066 children aged 4 years	55 (5.2)	40 (3.8)	4 (0.4)
Of 3671 children aged 5 years	213 (5.8)	84 (2.3)	12 (0.3)
Of 3286 children aged 6–7 years	167 (5.1)	46 (1.4)	3 (0.1)
**Mean test duration (SD), sec^b^**	66.8 (62.3)	91.8 (51.9)	158.6 (85.9)
Of those who passed	59.2 (44.2)	91.2 (50.2)	149.3 (69.4)
Of those who failed	200.2 (136.9)	109.0 (86.6)	323.9 (172.1)
**No. (%) of those who failed immediate rescreen and attended community-based second screen**	389 (89.4)	NA	NA
**No. (%) of those who failed community-based second screen**	124 (31.9)	NA	NA
**No. (%) of total who received diagnostic referral**	124 (1.5)	170 (2.1)^a^	19 (0.2)
**No. (%) who attended referral**	94 (75.8)	109 (64.1)^c^	9 (47.4)
**No. (%) of total with confirmed diagnosis**	54 (0.7)^d^	55 (0.7)^e^	2 (0.02)^f^

mHealth: mobile health technology; NA: not applicable; SD: standard deviation.

^a^ This number includes 123 children who failed the immediate rescreen plus 47 children who were erroneously not rescreened.

^b^ Initial screen duration for vision; combined initial and immediate rescreen for hearing.

^c^ 21 awaiting appointment.

^d^ 5 awaiting confirmation.

^e^ 8 awaiting confirmation.

^f^ 11 awaiting confirmation.

mHealth: mobile health technology

mHealth: mobile health technology

**Table 2:** Cost of screening for hearing and visual impairment via mHealth-supported community-based programme, South Africa, September 2017–December 2018

Service or goods	US$		
	Total cost for progamme^a^	Cost per month	Cost per child^b^
Mobile testing devices (four hardware sets)	4 163.78	260.24	0.52
Software (hearScreen, Peek Acuity)	4 404.80	275.30	0.55
Device calibration	499.69	31.23	0.06
Telecommunication	1 432.00	89.50	0.18
Salaries of CHWs (three screeners)	14 604.16	912.76	1.82
Salaries of CHW (both project administrator and screener)	9 759.04	609.94	1.22
Project management (including delivery of training course to CHWs)	3 560.32	222.52	0.44
Travelling (2.77 Rand per km)^c^	4 243.84	265.24	0.53
Administration	1 545.60	96.60	0.19
Programme resources (stationary, power banks, posters)	968.80	60.55	0.12
**Total**	**45 182.03**	**2 823.88**	**5.63**

CHW: community health worker; mHealth: mobile health technology; US$: United States dollars.

^a^ Programme was running over 16 months.

^b^ Total number of children was 8023.

^c^ In April 2019, 1 South Africa Rand is equivalent to US$ 0.069.

The number of children who failed the initial screen and rescreen was 435 (5.4%) and 170 (2.1%) for hearing and vision, respectively (Table 1). Hearing test failure was associated with longer test duration (odds ratio, OR: 1.022; 95% confidence interval, CI: 1.021–1.024) and noise levels exceeding maximum permissible ambient noise levels at the 1 kHz test frequency (e.g. for left ear, OR: 1.688; 95% CI: 1.198–2.377; Table 3), but not with sex (OR: 0.891; 95% CI: 0.702–1.131). CHWs failed to perform an immediate vision rescreen for 47 children and these children were assumed to have failed. Vision test failure was associated with a younger age (OR: 0.629; 95% CI: 0.520–0.761) and longer test duration (OR: 1.003; 95% CI: 1.002–1.005), but not with sex (OR: 0.928; 95% CI: 0.726–1.186). Mean initial test duration for children who passed the screening was 59.2 and 91.2 seconds for hearing and vision, respectively (Table 1).

**Table 3:** Maximum permissible ambient noise levels being exceeded at different test frequencies during hearing screening, South Africa, September 2017–December 2018

Ear	MPANL’s exceeded during screening *n* = 8023							
	1 kHz			2 kHz			4 kHz	
	No. (%)	OR (95% CI)		No. (%)	OR (95% CI)		No. (%)	OR (95% CI)
Left	2816 (35.1)	1.688 (1.198–2.377)		144 (1.8)	1.772 (0.510–6.162)		80 (1.0)	0.534 (0.156–1.821)
Right	2808 (35.0)	2.770 (1.931–3.974)		128 (1.6)	1.835 (0.482–6.988)		88 (1.1)	1.790 (0.307–10.427)

CI: confidence interval; kHz: kilohertz; OR: odds ratio; MPANL: maximum permissible ambient noise level.

Of the 389 children who attended a second hearing screening, 124 (31.9%) failed the hearing test again and were referred for a diagnostic evaluation (Table 1). Of the 265 children who passed the second hearing screening, the audiologist referred 66 (24.9%) for wax removal at their local clinic. Of the 94 children who attended a diagnostic referral appointment, 54 (43.5%) were diagnosed with a hearing impairment and nine (7.3%) were discharged from audiology, but referred for other developmental interventions; another five children have follow-up appointments to confirm hearing status (Table 1).

A total of 55 children were diagnosed with a visual impairment; however, 21 children were still awaiting diagnostic optometry appointments at the time of reporting (Table 1). Of the 8023 children screened, 111 (1.4%) were confirmed with either a hearing or visual impairment, or both.

## Discussion

Our mHealth-supported community-based hearing and visual screening programme was successful in several ways. The programme had a low cost of screening per child, high participation numbers, high attendance of those who failed initial screening and immediate rescreening at the community-based second screening and overall low proportion of children receiving a diagnostic referral to a public health institution. The programme encountered several challenges, such as CHW safety, logistics and technology, for which we developed mitigation strategies (Box 1).

Box 1 **Challenges and mitigating strategies of mHealth-supported community-based programme, South Africa, September 2017–December 2018**

• Safety in community: link to CHW WhatsApp group, with warnings about protests or high-risk areas to avoid on certain days; considering the cultural hierarchy, one CHW was a male.

• Safety of equipment: arrangements were made at the local clinic to safely lock away equipment overnight.

• Charging equipment: CHWs charged power banks at home and then used to charge devices overnight.

• Noise levels in preschool centres: (i) mHealth monitored noise for quality control; (ii) tests were conducted in neighbours’ homes if the centre was too noisy, involving the community further; and (iii) future protocol for high-noise settings will involve screening at 30 dB (instead of 25 dB) hearing level at 1 kHz.

• Absenteeism: (i) project administrator telephoned the preschool centre principal in advance to inform parents that children should attend on that day; (ii) staff fetched children from home or telephoned parents to bring children; and (iii) school and cultural holidays were avoided for screening, but used for CHW training and administration.

• Travelling in community: the implementation partner (Carel du Toit Centre) provided a car allocated to community outreach for CHWs to use.

• Language diversity: we appointed a diverse team of CHWs from the communities who could speak local languages.

• Informed consent: we provided a simplified single-page consent form in multiple languages, as well as the option for parents to send a free text requesting a call from the project administrator.

• Diagnostic follow-up attendance: parents were reminded of diagnostic appointments by telephone the week before the appointment, with the CHW emphasizing the importance of attendance, in the parents’ native language.

• Technology: (i) CHWs informed the project manager of problems; (ii) we held retraining and problem solving during weekly meetings; and (iii) we reported challenges and suggestions to hearX Group for developers to consider.

CHW: community health worker; db: decibel; hearx Group: mHealth technology platform; khz: kilohertz; mHealth: mobile health technology.

**Fig. 3:**
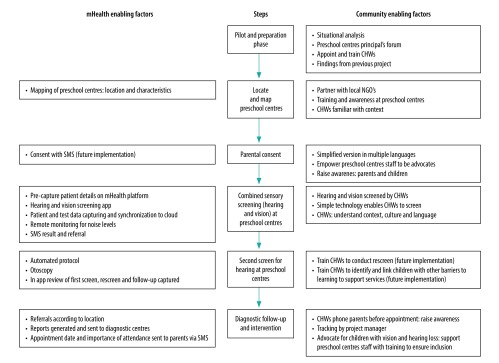
Enabling factors of service-delivery model for hearing and vision care for preschool children, South Africa

Use of the same equipment and minimally trained staff to screen both hearing and vision contributed to the affordability and scalability of the service-delivery model (Fig. 3).^13^^,^^14^^,^^23^ The low cost per child for dual screening reported in this study (Table 2) could be reduced further as CHWs continue to gain experience and efficiencies are increased.

App: smartphone application; CHW: community health worker; mHealth: mobile health technology; NGOs: nongovernmental organizations; SMS: short message service.

Employing CHWs from the community was invaluable for raising awareness with preschool centre staff and parents.^12^^,^^14^^,^^22^^,^^30^ Selecting communities where an existing public health pathway to intervention was already in place was another important factor contributing to the success of the model.^31^^,^^32^ A high informed consent return was supported by strong community involvement and the provision of simplified forms in local languages. The consent return could be further improved through a free text messaging service (Fig. 3). 

Locating the second screening for hearing impairment at the respective preschool centre yielded a high proportion of attendance compared with an earlier project in which rescreening took place at public health care institutions (89.4% versus 39.4%).^14^ Although an improved hearing test failure rate was achieved from initial screening and rescreen by CHWs (435/8023, 5.4%) to second screening by audiologist (124/8023, 1.5%), with further training, this second screening could also be conducted by CHWs to reduce the costs further. By achieving a final overall proportion of 1.5% for hearing impairment referral, our programme reduced the number of referrals to resource-constrained public health institutions.^14^^,^^23^^,^^29^^,^^33^ We hypothesize that the high proportion of diagnostic appointment attendance (75.8%) was attributable to the early confirmation of initial screening results, reducing the amount of follow-up appointments,^14^^,^^34^ and the use of reminders sent to parents.^35^

We identified background noise levels as a significant influence of screening outcome. Most of the failed hearing tests at which background noise levels were excessive (5624/6064, 92.8%) were recorded at the lowest pure tone test frequency (1 kHz); this issue could be addressed by increasing the hearing level (from 25 to 30 dB) to minimize noise interference at this test frequency.^14^^,^^15^^,^^23^^,^^24^^,^^36^

Mean test duration for hearing screening (combined initial and immediate rescreen time) was shorter than for a previous study (66.8 versus 177.8 sec),^14^ because hearing level was only rescreened at frequencies failed in the initial screening. Longer test durations were associated with failed screening outcomes for both hearing and vision; this is because more test trials were required for true positives. Longer test durations associated with false positives were because of poor comprehension of instructions and delayed or incorrect responses.^14^

The importance of an automatically initiated rescreen (included for hearing but not visual screening) was highlighted by the fact that 47 children were not immediately rescreened for vision due to tester error.^14^^,^^36^ Age did not affect results for hearing screening, but vision failure rates were twice as high in children aged 4 years compared with children aged 6–7 years, possibly because of a lack of comprehension or attention.^37^

Our observed prevalence of hearing (0.7%) and visual (0.7%) impairments was lower than the previously published estimates for young children of 2.4% and 3.9%, respectively.^6^^,^^7^^,^^13^ This might be because children with impairments are potentially less likely to attend a preschool centre, are still awaiting confirmation of status or, in the case of more severe impairments, have already been identified and are attending impairment-specific programmes. We could not find other published results with which to compare our observed prevalence of dual sensory problems. Although small, this prevalence highlights the importance of screening for both hearing and visual impairment; identifying an impairment in one modality does not predispose or preclude an impairment in the other.

Our study had limitations. No ophthalmic supervision was provided to CHWs and no measure of the quality of CHWs was available. A control group would have been valuable. The resource constraints in low- and middle-income countries were highlighted by the number of children still awaiting appointments at the end of the study period.^9^^–^^11^

Children with disabilities in LMICs are often unsupported without timely detection.^9^ In accordance with the leave no one behind movement that supports the sustainable development goals,^5^^,^^38^ we have shown that a decentralized mHealth-supported service-delivery system can provide increased access to hearing and vision services for preschool children in poor communities. Efficient design of such a system requires a holistic approach, including the use of digital technology, the training and monitoring of CHWs, the support of community partners and effective referral systems.

Future research should focus on evaluating the cost–effectiveness and impact of detection and intervention on educational and psychosocial outcomes; the perceived acceptability of such screening programmes to parents and caregivers; and the potential integration of other mHealth services, for example, developmental delay screening,^39^ towards a more comprehensive community-based service.
